# Is there any importance of Migraine with Aura amongst medical students?

**DOI:** 10.51866/lte.249

**Published:** 2022-10-17

**Authors:** Amar Taksande

**Affiliations:** 1 MD, FIAE, Professor, Deptt. of Pediatrics, JNMC, DMIMS, SawangiMeghe, Wardha, Maharastra, India. Email: amar.taksande@gmail.com

**Keywords:** Migraine, Aura, Medical students

**Dear Editor,**

I have read the article by Thiagarajan et al. which is highly informative and describes the profile of headache and migraine amongst medical students. However, there are a few points that require clarification.^1^

First, medical students aged ≥18 years who experienced headache in the previous 3 months were included in the study. According to Galinovi et al.,^2^ the prevalence of migraine was 8.86% in first-year students and 10.90% in sixth-year students, while the prevalence of tension headache was 60.13% and 57.69%, respectively. In the study, whether the prevalence of migraine and nonmigraine headache differs according to the year of study was unclear.

Second, a migraine can last between 4 and 72 hours. It can be difficult to predict how long a migraine will last; however, tracking its progress may be beneficial. Xie et al. recorded the duration of headache as the average number of hours for a typical headache attack and found that the average duration of a migraine attack was 3 (interquartile range: 1–4) hours.^3^ In the study, the authors did not indicate how long headache lasted amongst the medical students (2022).

Third, the most potent and consistent risk factor for migraine is a family history of this condition itself. Birkie et al. discovered that the risk of migraine increased by more than 3.83 times (adjusted odds ratio=3.83, 95% confidence interval=2.313–6.366) in the presence of a family history of headache compared with that in the absence of a family history of migraine.^4^ In the study, whether there was any correlation between a family history and the prevalence of migraine amongst the medical students was not reported.

Fourth, migraine aura symptoms include brief visual or auditory disturbances that occur prior to other migraine symptoms, such as severe headache, nausea and sensitivity to light and sound. The International Classification of Headache Disorders (ICHD) systematised the diagnosis of migraine with aura and migraine with typical aura for the first time in 1988, with updated criteria published in the second edition in 2004 and the third edition in 2018.^5^ In the cross-sectional study conducted in 2013, the authors used the ICHD diagnostic criteria to diagnose a possible migraine. However, they did not describe the migraine aura symptoms amongst the medical students.
